# Revitalizing preschool minds: a fresh approach to arts-based brain health interventions

**DOI:** 10.3389/fpubh.2024.1398925

**Published:** 2024-11-27

**Authors:** Joyce Siette, Emily Anderson, Michelle Catanzaro

**Affiliations:** ^1^The MARCS Institute for Brain, Behaviour and Development, Western Sydney University, Westmead, NSW, Australia; ^2^Design, School of Humanities and Communication Arts, Western Sydney University, Rydalmere, NSW, Australia

**Keywords:** dementia, brain health, literacy, preschool, parent

## Abstract

**Introduction:**

Brain health interventions seek to cultivate enduring, health-promoting behaviors for an enhanced quality of life. Despite this objective, achieving sustained adoption and engagement of healthy lifestyle behaviors remains a challenge, prompting the exploration of innovative avenues for promoting brain health. The potential of integrating brain health education in younger populations is particularly promising, given the likelihood of these habits persisting into adulthood. This study thus aimed to identify parental perspectives on a performing arts-based brain health intervention for implementation in preschool settings.

**Methods:**

Preschool parents and early learning center staff participated in three focus co-design groups (*n* = 12) to create a prototypical performing arts brain health intervention. Each focus group was recorded, transcribed and analyzed using deductive thematic analysis.

**Results:**

Three main themes for effective brain health interventions in preschool settings were identified: optimizing logistical processes to ensure efficient delivery and scalability, recognizing motivation as a central factor influencing sustained engagement across all stakeholders, and tailoring educational content with specialized explanations for children to enhance comprehension and relatability. A new implementation approach was proposed to support an arts-based brain health intervention designed for preschools.

**Conclusions:**

These findings have important implications for the future of brain health education focusing on youth populations and a lifelong approach to dementia risk prevention strategies. By addressing logistical challenges, prioritizing motivation, and tailoring explanations to the unique needs of children, future programs can be more adaptable and successful in fostering positive and sustainable brain health behaviors.

## 1 Introduction

Dementia consists of a group of neurological disorders impacting memory, cognition, and daily functioning (1). Over 55 million people are living with dementia worldwide, of which older individuals in lower and middle income countries are mainly impacted (2). Dementia poses a global public concern, with a predicted rise in affected individuals and awareness of more cases beginning in individuals aged below 65 (34). While there is no cure for dementia (2), raising awareness of risk reduction strategies remains essential (3).

Modifiable lifestyle factors play a pivotal role in cognitive and development, such that primary prevention strategies are essential for dementia risk reduction (4). Indeed, Livingston et al. (5) highlighted several modifiable risk factors for dementia, with the potential to prevent or delay onset in up to 40% of cases. Physical inactivity, poor diet, poor sleep hygiene, low social engagement, and poor mental health are of particular interest. However, current brain health and dementia risk reduction research predominantly focuses on older populations, which can hinder the adoption of risk reduction strategies in the long-term (3, 6–9). Targeting older populations within risk reduction strategies also does not address the increase in cases for individuals aged 65 and under.

The implementation of proactive strategies would address rising dementia rates. Targeting younger populations for primary dementia prevention and brain health promotion aligns with the likelihood of behaviors introduced in youth persisting into adulthood (10). A school-based dementia education program by Smith et al. (11) was able to raise knowledge and attitudes of dementia up to 6 months after the intervention's implementation. This success, coupled with the efficacy demonstrated by established play-based approaches like Crunch&Sip^®^ (12) and Munch and Move (13, 14), indicates the potential of engaging young minds to instill healthier lifestyles.

Parents and early learning educators play influential roles in shaping children's health behaviors (15, 16). Their active involvement is fundamental in delivering age-appropriate brain health education and assists with establishing a foundation for cognitive wellbeing from an early age. Furthermore, the non-stigmatized and inclusive environment provided by early learning center (ELC) staff (17) complements the broader impact of delivering brain health education in school settings which can build awareness and contribute to the creation of dementia-friendly communities (18).

Arts-based mediums, particularly in the performing arts, has attracted considerable attention for their potential to promote brain health across diverse populations and life stages (4, 19). These mediums are uniquely positioned to transcend cultural, linguistic, and age-related barriers, and offer potentially more inclusive frameworks by targeting and enabling cognitive and emotional wellbeing. Numerous studies, such as those by Greenfader et al. (20) and Foster and Jenkins (21), have demonstrated the capacity of performing arts-based interventions to support youth-centered healthy development. These interventions, spanning from early childhood to pre-adolescence, typically engage participants in activities that not only promote cognitive growth but also facilitate social relatedness, identity formation and a lasting enthusiasm for learning—all key contributions to long-term psychological and social wellbeing.

It is important to distinguish, however, that not all arts-based activities exert uniform effects on brain health. The type of artistic medium and the developmental stage of the participant can influence health and educational outcomes. For instance, performing arts such as drama and music may offer greater benefits for cognitive and social development by encouraging collaboration, empathy and self-expression, particularly during adolescence, a time of heightened identity exploration. In contrast, physical forms of artistic engagement, such as dance, have been found to more directly impact health and socioemotional development, particularly in early to middle childhoold.

Archbell et al. (22) and Thomaidou et al. (23) illustrate this by showing that dance interventions can enhance motor coordination, social connectedness, and emotional regulation during key developmental windows. Furthermore, McCrary et al. (24) highlight the broad spectrum of benefits conferred by performing arts-based interventions when integrated with regular, structured activities. Their review of health promotion programs that used expressive forms of dance, drumming, and aerobic dance across various age groups showed improvements in cognitive function, mental health, body composition, immune function, physical fitness, and social wellness. Such outcomes align with broader determinants of health, including physical activity, social engagement and psychological resilience, all of which contribute to holistic brain health. Thus, while there is evidence of the potential efficacy and multifaceted benefits of arts-based approaches, the specific type and timing of interventions are factors that modulate their impact.

Beyond the arts themselves, the integration of co-design methodologies into intervention development will ensure programs are contextually relevant and participant-driven. Co-design allows for the authentic, collaborative exploration of both barriers and facilitators and ensures that the interventions are grounded in the lived experiences of the target population (25–27). This participatory framework will enhance the cultural and social relevance of future programs, and at the same time, aligns with the concept of autonomous motivation (28). By encouraging intrinsic and well-integrated extrinsic motivation, co-design approaches can cultivate a sense of ownership, competence, and autonomy among participants, which will increase the likelihood of sustained engagement and intervention uptake over time. Therefore, performing arts may serve as a vehicle for cognitive and socioemotional growth in children, but their success is contingent upon the strategic integration of co-design principles that empower participants and tailor interventions to the unique needs and preferences of the communities these arts-based programs seek to engage.

This preliminary study thus aimed to gain insight on how to best design a performing arts-based brain health intervention for preschoolers, using a co-design methodology with preschool parents and early learning center staff to enable a life course approach toward brain health education.

## 2 Methods

### 2.1 Study design

A co-design workshop methodology using semi-structured focus groups with open-ended discussion questions was adopted. Each focus group completed three sessions of 1 h-long workshops over 3 weeks, with the final workshop resulting in a prototype performing arts-based intervention for promoting brain health in preschools. The study received ethics approval from the Western Sydney University Human Research Ethics Committee (reference number: H15440).

### 2.2 Participants

Participants, including parents of preschool aged children (three to 5 years) and early learning center (ELC) staff in Australia, were recruited through various channels such as flier advertisements, existing research databases, researcher networks, mailing lists, and word-of-mouth. Eligibility criteria required participants to be over 18 years old, able to communicate in English, and willing to provide extended informed consent. Exclusion criteria were applied if participants did not meet these requirements. To incentivise participation, a financial incentive of a $20 Preezee gift voucher was offered to each participant upon the completion of a focus group.

### 2.3 Materials and procedure

#### 2.3.1 Demographics survey

The parent demographics questionnaire included nine questions relating to personal characteristics and information regarding their children. Questions asked about age, gender, country of birth, highest level of education achieved, employment status, marital status, number of children, children's ages, and the location of their child's preschool. ELC staff completed the same demographics questionnaire with an additional question on employment details (e.g., employment status, role/title, location of the preschool they work at, qualifications related to their role, years in role, and number of children in their current care at work).

#### 2.3.2 Workshops

All participants completed three workshops over three consecutive weeks. Participants were organized into four focus groups based on their availability. Group size ranged from two-four participants. Workshops were conducted over Zoom (Version 5.15.7), with the audio of each recorded using Zoom's recording feature. Chat box content was also obtained.

##### 2.3.2.1 Workshop one

The first workshop began with a preamble outlining the importance of brain health, an introduction to the five modifiable risk factors (i.e., diet, sleep, physical activity, social engagement, and mental wellbeing), and the benefits of arts-based and co-design interventions. Participants were asked questions regarding their thoughts on brain health, potential barriers for implementing a performing arts intervention in preschools, and factors boosting their children's engagement. An example is “In what ways could we use performing arts to promote the five brain health behaviors?”

##### 2.3.2.2 Workshop two

The second workshop began with a summary of the previous workshop's discussion. Participants were shown several resources of performing arts activities paired with one of the five modifiable brain health factors. Examples include a healthy eating sing-along song and a dance follow-along for physical activity. Participants were asked questions about these resources regarding their likes, concerns, and their own ideas for structuring an intervention. An example is “What problems for implementation could you predict with the provided examples?” The Whiteboard feature on Zoom was implemented to summarize discussion content visually for clarity. This was used by the researcher to create a prototype intervention for discussion within the third workshop.

##### 2.3.2.3 Workshop three

The third workshop involved the researcher presenting the prototype intervention to participants based on the ideas discussed in the first two workshops. Participants were given the opportunity to express what they liked, what they wanted to change, and their overall thoughts on the creation process. An example is “Is there anything you would change about the program? If yes, what?” The Whiteboard feature on Zoom was used again to highlight any additional concerns, ideas, or changes raised by participants. Participants were also asked the rate their likelihood of using this intervention with either their children (parents) or within their preschool (ELC staff) on a 7-point Likert scale (1 = not likely to at all, 7 = highly likely to).

### 2.4 Analysis

The audio of each workshop was transcribed by the researcher using the dictation feature on Microsoft Word (Version 16.77.1). NVivo (Version 14.23.0) was used for data analysis. Workshop transcripts were independently analyzed using Braun and Clarke's (29) six-step approach to thematic deductive analysis by two researchers (JS and EA). In the first step, data was familiarized through an initial active reading, re-reading, and note-taking. This was to gain an overall understanding of the ideas highlighted across groups. The second step involved the systematic development of the initial codes. Some examples of these initial codes include “customisable program”, “visual aids develop skills”, and “modeling behaviors”.

The third step included collating initial codes into potential themes, centered around a primary code. An example of this is the primary code of “the individualized child”, including subcodes such as “child interests”, “child learning styles”, and “differently abled children”. These potential themes were revised in step four, in relation to the whole dataset and research question. Potential themes from the third step were merged, such as “Communicating Simply and Appropriately” and “Individual Child Differences” to form “Teaching Brain Health to Children”. Themes were then further refined, defined, and named during the fifth step, and verified within the research team.

## 3 Results

### 3.1 Overview

Adults (*N* = 12) participated in this study with a mean age of 37.33 years (SD = 5.09, range = 25–47) (Tables 1, 2). Parents (*N* = 8) were all female, with a mean age of 38.13 years (SD = 4.14). Most children attended preschool in the Greater Sydney region and their age ranged between 2 and 11 years. Four ELC educators were recruited (*n* = 4), were all female, with a mean age of 35.75 years (SD = 6.30) and a mean 8.5 years (SD = 6.69) spent in their role. All educators had received their Diploma in Early Childhood Education.

**Table 1:** Participant demographics for parents.

**Variable**	***N (%)***
**Country of birth**	
Australia	4 (50)
China	1 (12.5)
Taiwan	1 (12.5)
Colombia	1 (12.5)
England	1 (12.5)
**Highest level of education**	
TAFE	1 (12.5)
Bachelors	4 (50)
Masters	3 (37.5)
**Employment status**	
Full-time	5 (62.5)
Part-time	1 (12.5)
Sole trader	1 (12.5)
Non-employed	1 (12.5)
**Number of children**	
1	2 (25)
2	5 (62.5)
3	1 (12.5)

**Table 2:** Participant demographics for ELC staff.

**Variable**	***N (%)***
**Country of birth**	
Australia	2 (50)
India	1 (25)
Ecuador	1 (25)
**Highest level of education**	
TAFE	2 (50)
Bachelors	1 (25)
Masters	1 (25)
**Employment status**	
Casual	1 (25)
Part-time	1 (25)
Full-time	2 (50)
**Number of children in care**	
< 20	2 (50)
>20	2 (50)

**Figure 1:**
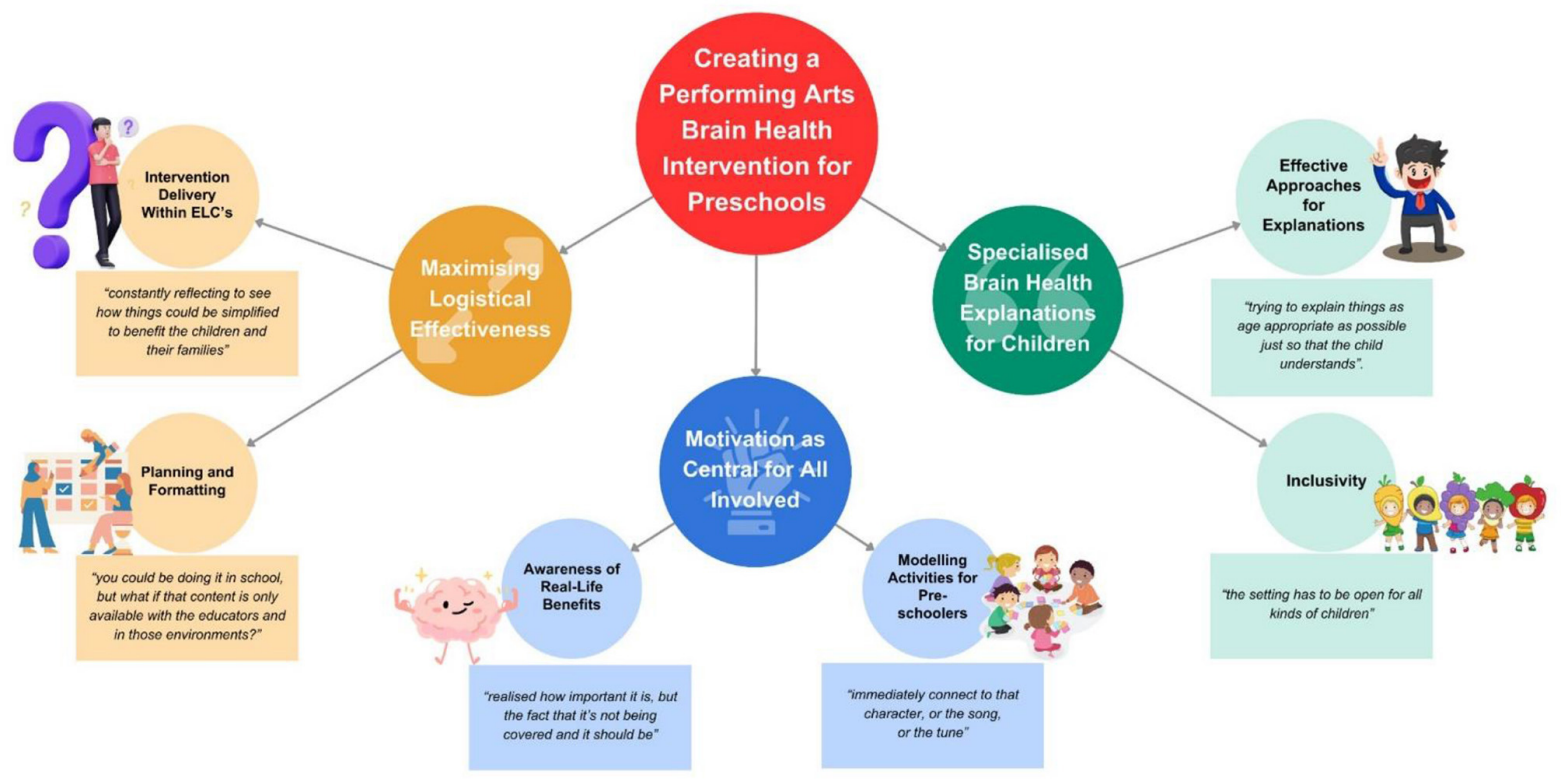
Visual representation of themes and subthemes.

Thematic analysis produced three primary themes, labeled “Maximizing Logistical Effectiveness”, “Motivation as Central for All Involved”, and “Specialized Brain Health Explanations for Children”. These themes are presented visually in Figure 1, with their respective subthemes.

### 3.2 Theme 1: maximizing logistical effectiveness

This theme highlighted the ongoing attention that parents and educators devoted to meeting the needs of their preschoolers, especially when brain health was concerned. Parents provided both positive and negative appraisals of this hypothetical program, and strengths and barriers were assessed through the lens of the most effective implementation within preschool settings. This was demonstrated through two subthemes: “intervention delivery within ELCs”, and “planning and formatting”.

#### 3.2.1 Intervention delivery within ELCs

Both parents and educators expressed optimism about the feasibility of incorporating a brain health intervention in ELCs and believed it could be done with the right approach. This optimism was grounded in the perception that brain health education aligns with existing curriculum content, particularly regarding health and safety, making it more compatible with ELC educational goals. This was supported by Educator 1 claiming “*it's certainly not impossible”* and was further backed up by Educator 2 stating that educators are “*happy to implement it [a brain health intervention] as long as it falls within the EYLF [Early Years Learning Framework] and that it is appropriate for that children's age group and their development”*.

Emphasis was placed on the busy nature of ELCs, suggesting that incorporating an extensive intervention may prove difficult and cause logistical challenges with scheduling. Educator 1 explained this challenge, stating it would be “*very difficult to fit all these lessons in throughout the week if you have to do one about Aboriginal perspectives, one of ‘your body is your body', and then another one for your healthy brain at scheduled times”*.

Parents and educators also raised concerns regarding the program cost, however their willingness to consider paying for these services demonstrated the perceived high value of brain health interventions in preschool education. Parent 1 stated that “*something like the healthy brain and mind is really important. I would be interested in paying for the services”*. Parents and ELC staff viewed delivering brain health interventions in ELCs as challenging but well within the realm of possibility.

The adaptive and reflective practice of educators was discussed as critical for developing, delivering and monitoring novel brain health resources and the program. Educator 2 explained how educators are “*constantly reflecting to see how things could be simplified to benefit the children and their families”*.

The educator's role was further broken down into significance of their delivery, where parents suggested that the best educators are both knowledgeable and engaging. Educator 1 used an example with their own child and stated that the “*teacher is so fun and engaging that the point gets across and he [the child] recalls it. Having an educator that is really passionate about it too, if you're passionate about it, they'll become passionate about it”*. Parents described the merits of using a fun and interactive approach to teaching their children, as this format typically received the best results for recall in the preschool age. Parent 2 supported this by stating that “*the main thing is just like having fun and feeling enthusiastic”*. The preference for knowledgeable educators suggested that there was a need to educate ELC staff and parents in brain health information prior to implementation. Educator 3 suggested that “*having little resources for the educator so they can also learn more about it”* could prepare educators for providing this education.

#### 3.2.2 Planning and formatting

Several resources were discussed to assess their applicability. Both parents and educators enjoyed dance and music as performing arts mediums, suggesting that these mediums are preferrable as they apply to a wide variety of children (differing ages, linguistic ability, and/or cultural background). Educator 2 stated that “*dance and music are largely appealing to a general group of children because it's very relatable to them”*. Books and technology were also popular for explaining important concepts to preschoolers, identified as potential resources to use within a brain health intervention. However, it was also noted that a large barrier to implementation was having access to these resources, especially for parents. Parent 3 voiced that “*you could be doing it in school, but what if that content is only available with the educators and in those environments?”* Technology was suggested as a solution, as parents and educators noted that having either a website or an app could connect a preschooler's school and home activities and increase parental resource access.

When discussing broad program effectiveness, repetition and routine-building were highly favored in both preschool and home settings. Educator 2 claimed that “*the more we do it [an activity], the better it gets into their minds. In fact, they fall into that routine very easily”*. Parents and ELC staff claimed that repetition solidified concepts for preschoolers and emphasized their importance, which helped to build consistency and knowledge for the children. However, concerns with routines and repetition were raised. Educator 3 claimed that “*not everyone is going to love it, not everyone's going to do it because not everyone is going to be into it”*.

Parents and educators strongly believed in the individuality of each child, and consistently brought up how children have different interests and learning styles. Offering variety and choice was emphasized. Parent 4 highlighted the importance of making activities feel non-compulsory to allow children to feel in control. They noted, “*it's not just like being told what to do, they are thinking about it for themselves…give them that choice”*. To prevent child boredom and disengagement, incorporating child-led moments and diverse activity formats, whether individually or in group settings, was recommended. This approach can help to cater to children's personalities and learning styles.

### 3.3 Theme 2: motivation as central for all involved

This theme demonstrated the primary role of motivation for preschool children, parents, and ELC staff in the success of a performing arts brain health intervention. Factors that boosted the motivation and engagement of each group were assessed to identify what facilitates intervention implementation. This was demonstrated through subthemes: “awareness of real-life benefits” and “modeling activities for preschoolers”.

#### 3.3.1 Awareness of real-life benefits

Awareness of brain health importance was not apparent for participants in the first workshop yet this awareness grew across the co-design process. Parent 4 highlighted that “*if I hadn't been in this focus group, I don't think I would know all of this stuff as a parent even”*. This was also demonstrated by Parent 1, identifying how they “*realized how important it is, but the fact that it's not being covered and it should be”*. This emphasized an increased importance of introducing brain health to preschoolers, to not only benefit the students, but also the parents and educators.

Parents and ELC were motivated by the idea of increasing their children's awareness of brain health with the hope that it could sustain healthy behaviors further into the child's life. Parent 5 supported this idea, stating that “*exposing them [the preschoolers] is always good”*.

Additionally, self-proclaimed “involved” parents and ELC staff were also motivated by the opportunity to learn more about brain health themselves through the interventions aimed at their children. Parent 3 shared that it was “*quite interesting to have these discussions and learn some of the things that you've been sharing”*. Parents and ELC staff conveyed an interest in engaging with brain health resources, which uncovered the motive to raise awareness in parents and educators as well as preschoolers.

#### 3.3.2 Modeling activities for preschoolers

The shortened attention spans of preschool-aged children were also highlighted, alongside consideration of which methodologies protected against this shortened attention. It was suggested that children love interactive, visual resources. Children also enjoyed resources when presented in short intervals during their morning time session. Modeling was highlighted as an efficient methodology that increased attention span associated with a particular activity. Educator 2 explained how children in their center “*immediately connect to that character, or the song, or the tune”*. Parents and educators also made links between the performing arts mediums and modeling techniques for capturing preschooler's attention for extended periods of time. Parent 4 gave a specific example of their children, stating that “*modeling after someone on the screen or the teachers”* was the most useful technique for child engagement and motivation.

### 3.4 Theme 3: specialized brain health explanations for children

This theme highlighted techniques used by parents and ELC staff to explain complex subjects to preschool aged children. Preferable explanations were described as general and simple for all children, but also as explanations that considered differing cultural backgrounds and neurodiversity. It encompassed two subthemes: “effective approaches for explanations” and “inclusivity.”

#### 3.4.1 Effective approaches for explanations

Both parents and educators recognized children's natural curiosity and their desire for broad learning experiences in both ELC and home settings. Open communication with children surrounding their learning was seen as important and can enable them to ask questions and explore. Parent 6 exemplified this, stating “*when I have little learning opportunities, I'll talk to him and I'll explain everything”*. Compassion and encouragement were suggested as vital components of these dialogues, which can make complex concepts feel normal. The content of these discussions also mattered, with Parent 1 reinforcing the importance of words used, “*I think to me the words are quite important, so I'd be interested to know what the actual words were”*.

Parents and educators also stressed the need for simple, age-appropriate explanations. Parent 3 questioned if it was “*possible to narrow it down even further”* when presented with the prototype brain health intervention in their third workshop and further raised the importance of simplicity for this age group. Parent 7 echoed this sentiment, stating that “*I would agree with trying to explain things as age appropriate as possible just so that the child understands”*. Visual resources paired with explanations were suggested to simplify brain health education for preschoolers, as it “*seems to get messages across a bit easier for them where they can interpret it and understand it and recall it better” (Educator 1)*. However, it was acknowledged that overly bright visuals could overstimulate children and hinder understanding.

#### 3.4.2 Inclusivity

Parents and educators had a strong belief that brain health education needed to be applicable to all kinds of preschoolers. Educator 2 conveyed that “*the setting has to be open for all kinds of children from different backgrounds, different needs, and different cultural requirements”*. This suggested the need for specific, yet simple explanations that extended to a wide variety of individuals.

Parents and educators found that arts-based mediums like performing arts mediums apply cross-culturally. Parent 4 supported this, stating that “*I think also for people who don't necessarily speak English, you can tell from the catchy tone that is a happy song…if they might not know the language, they can at least feel the vibe”*. This was echoed by Parent 2, who used songs and music to teach their daughter both Spanish and English vocabulary. Extending from this, an emphasis was placed on incorporating Aboriginal and Indigenous perspectives into brain health interventions, paying particular attention so “*we're not tokenistic about anything” (Educator 1)*. However, parents and educators still highlighted differing cultural backgrounds and linguistic abilities as potential barriers for implementing a brain health intervention, and suggested it was an aspect that needs to be continually considered.

When discussing differently abled children, parents and educators were mindful on “*how they [differently abled children] could be involved in the program without being made to feel different” (Educator 1)*. It was explained that the needs of differently abled children often led them to feel frustrated, comparing themselves to other children. The use of visual resources was heavily suggested to explain activities for differently abled children, especially for visually displaying the steps they need to take related to a particular activity/concept. Parent 2 suggested that “*popups or drawing something are better for them, less words or more images”*. This drew close ties with the implementation of arts-based activities for brain health education and highlighted the use of simple explanations. Performing arts were described as an effective medium for bringing the same experience to a differently abled child that a neurotypical child experiences.

### 3.5 Prototype intervention

**Figure 2:**
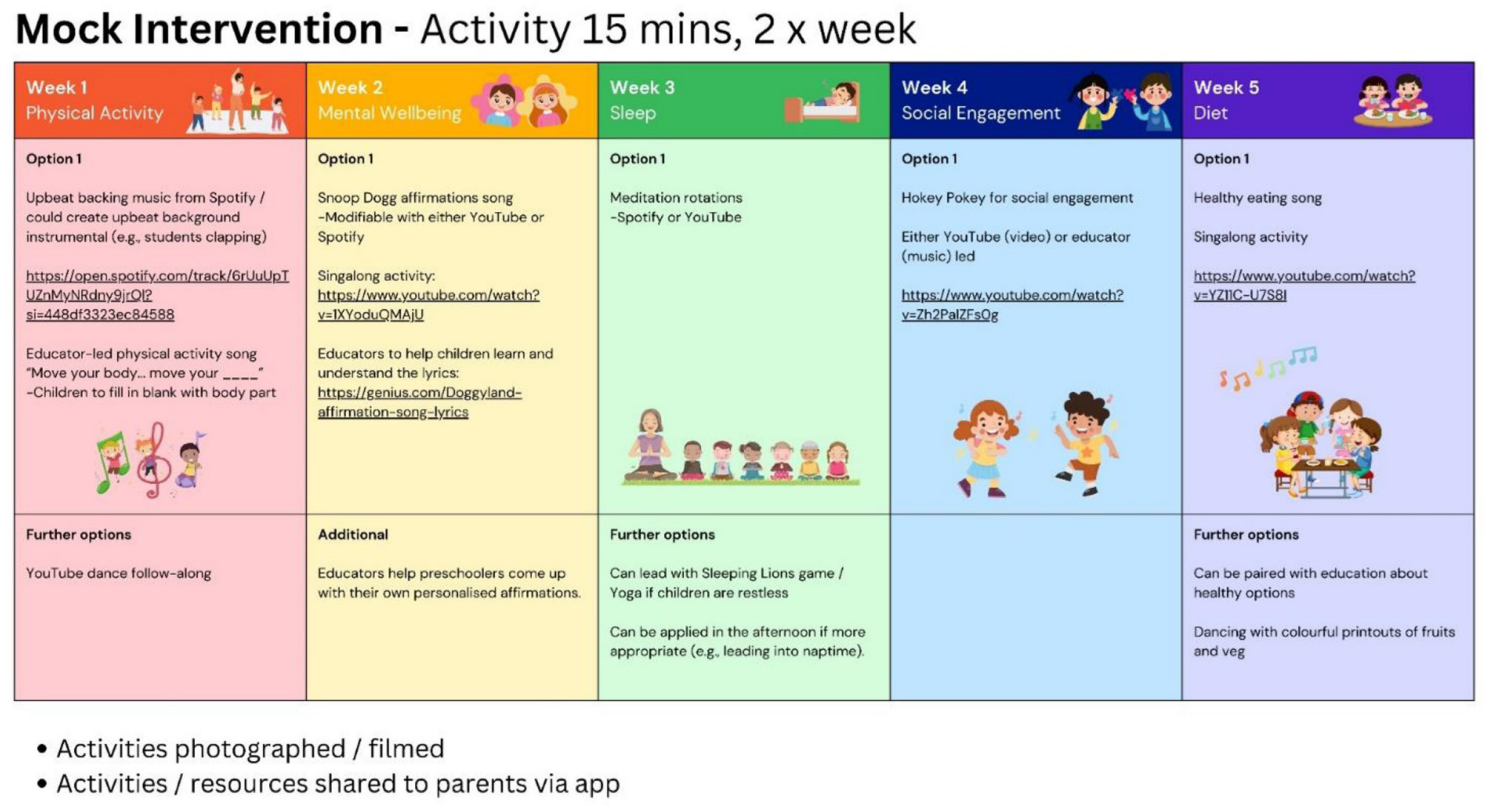
Example of a mock performance arts-based brain health education program.

A prototype of a performing arts brain health intervention for potential implementation within a preschool setting is shown in Figure 2. The intervention was broken into five weeks, each focused on a different modifiable factor for brain health. An example is week one, centered around healthy eating. Suggested activities included a healthy eating sing-along song, craft activities paired with dancing to a healthy eating song, and a roleplay of a grocery store selling fruits and vegetables, with these activities spread across the week for access to children who do not attend preschool every day. This continued for the following weeks with their respective modifiable factor. The group also suggested using an ELC app to share content, having a communal whiteboard to convey information, and emailing resources to parents. When rating the likelihood of using the prototype brain health program, most participants rated highly (Mean = 6.25 (out of 7), SD = 0.72), indicating that participants were likely to use the prototype intervention with their children.

## 4 Discussion

This study aimed to understand parental and educator perspectives on performance arts-based brain health educational preschool programs. Whilst parents and educators were willing to engage with brain health education, they discussed the need to create a holistic and well-coordinated approach to such interventions. Our findings suggest that successful programs should not only address logistical challenges but also prioritize strategies that boost motivation and incorporate specialized educational content for effective communication with children of diverse needs. Such an understanding can contribute to the refinement of existing implementation frameworks and strategies for optimizing the impact of brain health initiatives, and support promoting long-term behavioral changes and wellbeing across varied populations.

Parents and educators saw brain health education as useful and appropriate for preschool-aged children and agreed that the childhood period offers a unique window for brain health education to be sustained. These findings contribute to the growing research that brain health education in youth populations is an effective method for overall brain health promotion and awareness and supports the idea of primary prevention for dementia risk reduction in younger population groups (10, 14, 27).

Our study highlights the necessity for early childhood education programs to be underpinned by robust relationships characterized by engagement, communication, and active involvement among parents, ELC staff, and preschool students. Notably, parents and educators expressed the importance of flexible and adaptable brain health resources, integrating digital tools such as apps, to facilitate clear communication, interactive activities, and resource sharing. This aligns with the broader literature highlighting the pivotal role of strong relationships and effective communication between parents, educators, and young children in fostering optimal development during the formative years. Epstein and Sanders (30) raises the significance of family-school partnerships in supporting children's academic and socioemotional growth through collaborative efforts between parents and educators. Similarly, McNally and Slutsky (31) identified the key role of positive teacher-child relationships in early learning environments, emphasizing their contribution to children's cognitive and social development.

Moreover, the integration of digital tools into early childhood education corresponds with contemporary educational trends. Plowman and Stephen (32) and Takeuchi and Stevens (35) demonstrates the potential benefits of technology integration, suggesting that when implemented thoughtfully, digital tools can enhance communication, enrich learning experiences, and increase parental engagement. Our study's recognition of the importance of relationships, communication, and digital resources echoes these broader themes and accentuates the multifaceted nature of effective educational programming (33). Furthermore, our recommendation to incorporate flexible brain health resources, including digital tools for clear communication and shared resources, resonates with the evolving landscape of early childhood education (36), highlighting the imperative for collaborative efforts and innovative approaches to support young learners' development in the digital age.

The assertion that performing arts serves as an effective brain health education tool, supported by our study results, aligns with a growing body of literature on the potential of arts-based interventions in cognitive wellbeing. Several studies substantiate the notion that the visual and engaging nature of performing arts can enhance the efficacy of educational initiatives. Research by Greenfader et al. (20) illustrates the positive impact of a creative arts movement intervention on English-speaking skills, language comprehension, and social engagement in young children. Similarly, Foster and Jenkins (21) observed that children's participation in performance arts from ages 0–12 promoted social relatedness, identity formation, and lasting enthusiasm for learning, aligning with our findings.

Whilst the existing literature acknowledges positive outcomes from performing arts interventions, it further calls for a closer look at potential limitations. This includes reviewing how access to resources (digital tools and resources) and contextual factors (e.g., individual differences) in diverse educational settings (e.g., resource rich ELCs) influence the adoption and sustainability of such programs and the impact on benefits over time. Furthermore, additional research is required to compare the effectiveness of different forms of performing arts and explore the impact of cultural and socioeconomic factors on knowledge and health outcomes. This is essential for gaining deeper insights into how these factors differentially impact on children's cognitive and socioemotional development.

### 4.1 Strengths and limitations

Our study has several limitations, including the absence of preschool children's perspectives which hinders insights into their interests and comprehension of brain health. Future research should involve children in co-designing brain health interventions or conducting usability tests to gain their input. Additionally, the use of online workshops via Zoom may have affected participant engagement and depth of interaction. Virtual platforms can introduce challenges such as delays in sound transmission, varying image quality, and differences in internet connectivity may impede clear community and affect participants' perception and response to the proposed interventions. While this method was necessary due to logistical constraints, future studies should explore in-person workshops and determine whether these can elicit additional information. Another limitation of this study is the reduced sample size, although adequate for co-design methodology, our participant pool was exclusively female and the absence of male participants restricts the scope of the findings. Incorporating male perspectives in future research could provide a more holistic understanding of the barriers and enablers within home and ELC environments and provide a broader view of how gender dynamics might influence these contexts. Further, participants noted that success would rely on engaged relationships, noting that not all carers have the skills or interest in supporting the program. To address this, future studies can consider ways to incentivise educators, parents and students to increase participation (e.g., possibly through gamification or reward systems).

Positively, our study's methodology successfully gathered perspectives from both preschool parents and ELC staff, building a comprehensive understanding of effective strategies and impediments in conveying brain health information to preschoolers. The deliberate use of a co-design approach facilitated a mutually beneficial relationship between researchers and participants, promoting sustained engagement and comprehension of a hypothetical intervention over time. Moreover, the continuity observed across three workshops can support a sense of shared ownership, thereby augmenting brain health awareness and understanding among both parents and educators. Lastly, the study demonstrated inclusivity by incorporating participants from varied cultural backgrounds, ensuring a representative 50% inclusion of this diversity.

Despite these limitations, our study represents a preliminary approach and provides the groundwork for more extensive research. However, the findings should be interpreted with caution due to the aforementioned limitations, and future studies are recommended to validate and expand upon these initial results.

### 4.2 Future directions

Continuing research into brain health education for children is of the most significance. This study provides a promising, novel program that presents the opportunity for future research to adopt and test with different, diverse populations. A longitudinal study trialing a prototype performing arts-based brain health intervention with preschool students is a potential future research avenue that would provide direct feedback on the framework's success from the children themselves, improving its efficiency and engagement. This would further enhance the development of health psychology by promoting healthier lifestyles among preschool students, and further solidify the significance of investigating brain health education with a life course approach.

## 5 Conclusion

This study enhances our understanding of effective strategies for promoting brain health in children, particularly through arts-based approaches. The findings, coupled with the co-design methodology employed, offer new insights for developing future brain health educational programs.

## Data Availability

The datasets presented in this article are not readily available because due to the nature of qualitative data, only composite data is available upon reasonable request to the corresponding author. Requests to access the datasets should be directed to joyce.siette@westernsydney.edu.au.
